# In Situ Synthesis of Environmentally Friendly Waterborne Polyurethane Extended with Regenerated Cellulose Nanoparticles for Enhanced Mechanical Performances

**DOI:** 10.3390/polym15061541

**Published:** 2023-03-20

**Authors:** Soon Mo Choi, Soo Young Lee, Sunhee Lee, Sung Soo Han, Eun Joo Shin

**Affiliations:** 1 Research Institute of Cell Culture, Yeungnam University, 280 Daehak-ro, Gyeongsan 38541, Republic of Korea; 2 Department of Polymer Science & Engineering, Pusan National University, Busandaehak-ro 63 Beon-gil 2, Busan 46241, Republic of Korea; 3 Department of Fashion Design, Dong-A University, 37 Nakdong-daero 550 Beon-gil, Busan 49315, Republic of Korea; 4 School of Chemical Engineering, Yeungnam University, 280 Daehak-ro, Gyeongsan 38541, Republic of Korea; 5 Department of Chemical Engineering, Dong-A University, 37 Nakdong-daero 550 Beon-gil, Busan 49315, Republic of Korea

**Keywords:** stretchable, waterborne polyurethane, regenerated cellulose, nanocomposite film, natural filler, environmentally friendly

## Abstract

The development of waterborne polyurethane (WPU) has been stimulated as an alternative to solvent-based polyurethanes due to low-VOC alternatives and reduced exposure to solvents. However, their relatively low mechanical performance and degradation have presented challenges in their wide application. Here, we developed environmentally-friendly bio polyol-based WPU nanocomposite dispersions and films, and presented the optimal process conditions for their manufacture. Additionally, the condition was established without using harmful catalysts or ethyl methyl ketone (MEK) during the polymerization. Moreover, regenerated cellulose nanoparticles (RCNs) were employed as natural chain-extenders in order to improve the biodegradability and mechanical performances of the nanocomposite films. The RCNs have a lower crystallinity compared to cellulose nanocrystals (CNCs), allowing them to possess high toughness without interfering with the elastomeric properties of polyurethane. The prepared CWPU/RCNs nanocomposite films exhibited high toughness of 58.8 ± 3 kgf∙mm and elongation at break of 240 ± 20%. In addition, depending on the molar ratio of NCO/OH, the polyurethane particle size is variously controlled from 70 to 230 nm, enabling to fabricate their dispersions with various transmittances. We believe that our findings not only open a meaningful path toward green elastomers with biodegradability but provides the design concept for bio-elastomers in order to develop industrial elastomers with mechanical and thermal properties.

## 1. Introduction

Plastics are utilized in almost all fields in modern life; they are involved in packaging, furniture, clothes, bottles, electronics, vehicles, etc. Especially, these days, increased contact-free consumption due to COVID-19 has increased the use of plastics. A world without plastic has become unimaginable. Among them, polyurethane (PU) known as the fifth largest plastic has been routinely used as an organic material due to its relatively excellent performance, which is widely applicable in life [1,2,3]. However, since the 1990s, non-perishable plastics emerged as an environmental problem, and plastics including PU have become a symbol of environmental pollution. In addition, they can hardly be free from the issue of volatile organic compound emissions. The entire world has observed diverse environmental concerns related to plastics; progress has been accordingly accomplished towards the production and the employment of biodegradable plastics, which have structures allowing them to be easily broken down by microorganisms. However, biodegradable plastics have limitations in practicality due to difficulty in molding or poor physical properties [4,5,6]. Accordingly, the current trend has been progressively towards extensive research on waterborne polyurethane (WPU) as a replacement for solvent-borne polyurethanes (PUs) [7,8]. Waterborne polyurethane (WPU) has been substituted in existing PUs involved in the issue of organic solvents due to the increasing attention to health and environmental issues. Due to international limitations about the releasement of volatile organic compounds (VOCs) to the aerosphere, environmentally friendly products have recently become popular in various industries [9,10,11]. In the case of WPU currently being researched, not only petroleum-based diols, diisocyanates, and chain extenders, but also catalysts and viscosity modifiers harmful to the human body have been used as raw materials in the synthesis process, which are not environmentally friendly. In addition, the properties such as low mechanical strength and toughness of WPU are being discussed as main obstructions [12,13], and recently, research on the incorporation of fillers including graphite, carbon nanotubes (CNTs), and inorganic particles to improve them has been conducted [14,15,16].

Various vegetable-based oils are one of the alternatives to replace petroleum-based reagents in the synthesis of various polymers. Particularly, castor-oil is best option for the manufacture of Pus [6] owing to the hydroxyl groups residence in their structure enabling them to react with isocyanate groups for the urethane bond-forming with no pre-processing. There are benefits such as low toxicity and reusability in castor oil, in common with all vegetable oils, which in turn has led to draw attention on the synthesis of PUs and WBPUs (Waterborne Bio-based Polyurethanes) [17,18,19,20,21,22]. Castor oil-based WPU has a group of multiblock copolymer elastomers with alternating soft and rigid hard segments, this multiblock structure shows excellent flexibility and elasticity attributed from the soft segments. Hard segment domains are based on the role of reinforcing agents, pseudo-net, or physical crosslink points [23]. Nonetheless, the improvement related to their thermal and mechanical stability are still issues to be further discussed in depth [24,25]. In this study, we highlighted on the introduction of regenerated cellulose nanoparticles (RCNs) with low crystallinity produced from microcrystalline cellulose (MCC), as chain extenders to improve mechanical properties of natural based CWPU without interfering with its inherent characteristics. RCNs have been currently applied in many fields owing to their unique properties including biodegradability, biocompatibility, reusability light weight, and especially abundance. We already have expertise regarding the preparation of RCNs from NaOH-based solutions and urea and previously published findings about the properties of RCNs [9,26]. Finally, we prepared CWPU/RCNs nanocomposite films through synthesizing CWPU using bio-based oil and the incorporation of RCNs as a chain-extender. With the use of RCNs, the mechanical properties mentioned above could be improved while maintaining the unique elastic properties of CWPU. In addition, the purpose of this study is to use acetone as a viscosity regulator and RCNs as natural chain-extenders, without using harmful catalysts, which in turn developed CWPU/RCNs dispersions and films with good biodegradability and toughness. In addition, it was found how the properties of the resulting CWPU/RCNs nanocomposite films were affected depending on when RCNs are added. That is, we studied at which stage adding RCNs enhances the bonds between CWPU and RCNs. Furthermore, it was discussed how the mechanical, thermal, and bio-degradable properties of the fabricated CWPU/RCNs nanocomposites were altered according to the amount of RCNs used.

## 2. Materials and Methods

### 2.1. Materials

Castor oil, isophorone diisocyanate (IPDI), dimethylol butanoic acid (DMBA), and triethylamine (TEA) were purchased from Sigma-Aldrich Corporation (Yongin, Korea). Urea, NaOH, and other chemicals employed were all AR grade. Celluase enzyme acquired from Aspergillusniger was purchased from Sigma. *N*,*N*-dimethylformamide (Alfa Aesar, Karlsruhe, Germany) and Acetone (Junsei Chemical Co., Ltd., Tokyo, Japan) were used without further purification [22].

### 2.2. Preparation of RCNs

The preparation of regenerated cellulose nanoparticles (RCNs) was described in a previous paper. RCNs of aqueous dispersion with solid contents of about 0.96 ± 0.04 wt% were obtained. Five (5) g of MCC was blended with 100 mL NaOH 7% and urea 12% during 2 h at 25 °C, and then kept for 12 h at 220 °C. The mixture was kept at 4 °C after the dissolution of cellulose, after which the cellulose dissolved in the solution and was regenerated through the addition of 10 times *v/v* deionized water, followed by centrifugation at 5000 rpm for 10 min to precipitate and separate the regenerated cellulose. The precipitated cellulose was sonicated for 10 min at 80% (125 mm) amplitude (Vibra cell VC 130, Sonics & Mzterials, Inc., Newtown, CT, USA) after rinsing five times with deionized water in order to remove residual NaOH and urea. The solution was adjusted to pH 9 [22].

### 2.3. Preparation of CWPU and CWPU/RCNs Nanocomposite Dispersions and Films

**Scheme 1:**
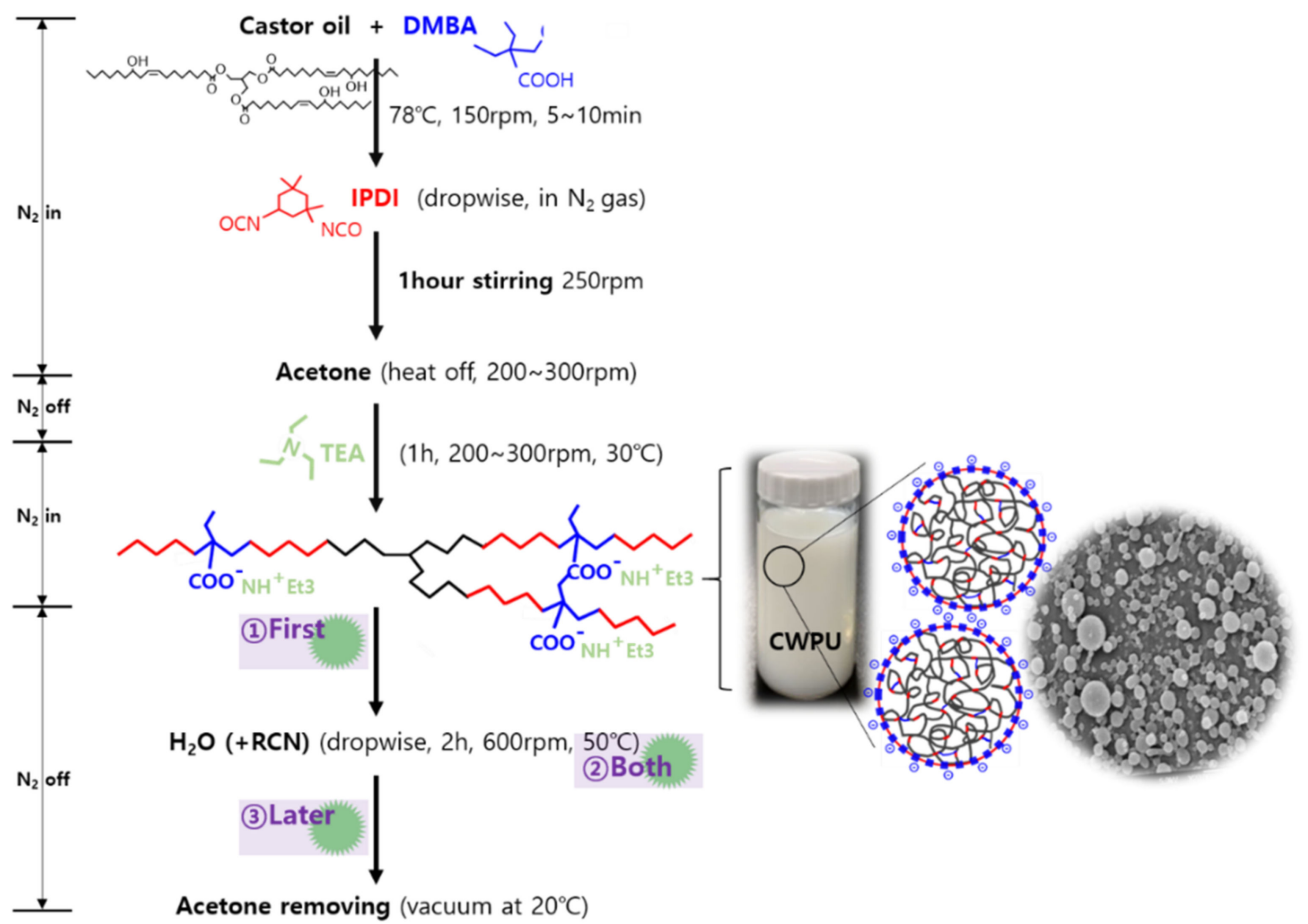
Synthetic process of CWPU/RCNs nanocomposites.

An acetone process was employed to control the CWPU prepolymer’s viscosity during the polymerization, because it was associated with obtaining a homogeneous solution, a diverse scope of structure, high quality as well as reproducibility of final products. Here, we also considered how the properties of the resulting nanocomposite films were affected depending on when RCNs were added. According to this concept, RCNs were added in three different steps with ① first (before the addition of water), ② both (with water), and ③ later (after the addition of water), during the CWPU synthesis process (Scheme 1), and the composition of CWPU/RCNs nanocomposites synthesis is also shown (Table 1). For the “① first”, which is a method of injecting RCNs before the addition of water, the RCNs were added before the CWPU nanoparticles were formed by water, and then water was added in the next step, which is coded as “CWPU/RCNs-first”. In this case, it is supposed that CWPU/RCNs nanocomposites are formed by hydrogen bonds of RCNs with PU chains prior to the formation of CWPU nanoparticles. For the “② both”, which is a method of injecting RCNs when adding water, the aqueous suspension of pre-diluted RCNs in water was added in the water adding step, which is coded as “CWPU/RCNs-both”. Finally, for the “③ later”, the RCNs dispersion was added after adding water, which coded as “CWPU/RCNs-later”. It means to add RCNs dispersion after the formation of CWPU nanoparticles; this mentioned method is employed for CWPU/RCNs nanocomposites when RCNs often play the role of chain-extenders. The main reaction for the formation of CWPU/RCNs involves the efficient crosslinking between CWPU prepolymer molecules with isocyanate groups and hydroxyl groups of RCNs as CWPU is complexed with RCNs. CWPU/RCNs nanocomposite films were fabricated via casting process. The CWPU/RCNs aqueous dispersions were sonicated during 1 h before casting and placed on glass square mold molding type by Teflon tape with 0.32 mm thickness. Then, it was dried in a chamber at 25 °C and 50% relative humidity for 24 h, and progressed in oven at 80 °C for 1 h and in vacuum oven at 40 °C for 24 h, respectively. The film‘s thickness for the tensile strength was 0.26 ± 0.03 mm, and ten samples were used for all analyses. 

### 2.4. Analysis of CWPU/RCNs Nanocomposites Dispersions and Films

Brookfield viscosity: all dispersion samples’ viscosity was conducted by a DV2T viscometer (AMETEK Brookfield, Middleborough, MA, USA). Analysis was carried out at 12, 30, or 60 rpm of the spindle at room temperature, respectively.

Zeta-potential and particle size distribution: the zeta potential, the average particle size, and the its distribution of the samples were analyzed using a Nano-ZS 90 zeta potential analyzer (Malvern Instrument Co., Ltd., Worcester, UK), respectively. The samples were added to a deionized water tank the pinhole of 200 μm and measured at 25 °C.

Fourier-transform infrared analyses (FT-IR): FTIR spectroscopy was monitored at 25 °C a Nicolet FTIR spectrometer (Thermo Nicolet Nexus 470 ESP FT-IR) (PerkinElmer, Inc., Waltham, MA, USA). The solidified RCNs and CWPU/RCNs were grinded with KBr and compressed to make pellets.

Nuclear Magnetic Resonance spectroscopy (NMR): all ^13^C NMR CWPU and CWPU/RCNs spectra were recorded on a Bruker AVANCE III^TM^ HD 400 MHz (Bruker Corporation, Billerica, MA, USA) and the use of a probe size of 4 mm. The spinning speed was 12 kHz, number of scans 16,384, relaxation delay 8 s, and acquisition time 12 ms. Approximately 10 mg of sample was packed in a sample holder.

Scanning electron microscopy (SEM): the morphologies of the CWPU and CWPU/RCNs nanocomposite films were examined by using a JSM-6400F scanning electron microscope (SEM) instrument (JEOL, Tokyo, Japan) operated at 5 kV accelerating voltage.

Thermogravimetric analysis (TGA): A thermogravimeter (TGA Q500, TA instruments, New Castle, DE, USA) was used to measure the weight loss of the RCNs, CWPU, and CWPU/RCNs nanocomposite. Ten (10) mg of the sample was heated from 30 to 600 °C with a heating rate of 10 °C/min.

Dynamic mechanical analysis (DMA): the dynamic mechanical thermal properties of film all samples were analyzed with 1 Hz using a DMA-Q800 (TA Instruments, New Castle, DE, USA) with a heating rate of 4 °C/min from −80 to 80 °C. The samples’ size was 20 mm × 6 mm × 0.2 mm for the DMA measurements. This characterization instrument was utilized at Core Research Support Center for Natural Products and Medical Materials (CRCNM) in Yeungnam University.

Tensile properties: the tensile strength was investigated by Autograph tester Instron 4201 (Shimadzu Corporation, Kyoto, Japan). The samples’ width and length were 5 mm and 20 mm, respectively, with dumbbell shape, respectively, and the test speed was set to 100 mm/min.

Enzymatic Hydrolysis: the CWPU and CWPU/RCNs films were transferred to 45 mL of citrate buffer (50 mM, pH 4.7) for enzymatic hydrolysis analysis. Cellulase enzyme was added and mixtures were allowed to undergo enzymatic hydrolysis at 50 °C under shaking, which is most optimum states for enzyme. The samples were decomposed over time, and the decomposition amount was calculated by following equation

(1) $$\mathrm{Decomposition}\;\mathrm{rate}\;(\%)=\frac{\mathrm{Wo}-\mathrm{Wt}}{\mathrm{Wo}}\times 100$$

where W_o_ and W_t_ are the weights of swollen films in citrated buffer solution and degraded films in citrated buffer with cellulase solution, respectively.

## 3. Results

### 3.1. Properties of CWPU Dispersions According to Emulsifier Ratio 

**Figure 1:**
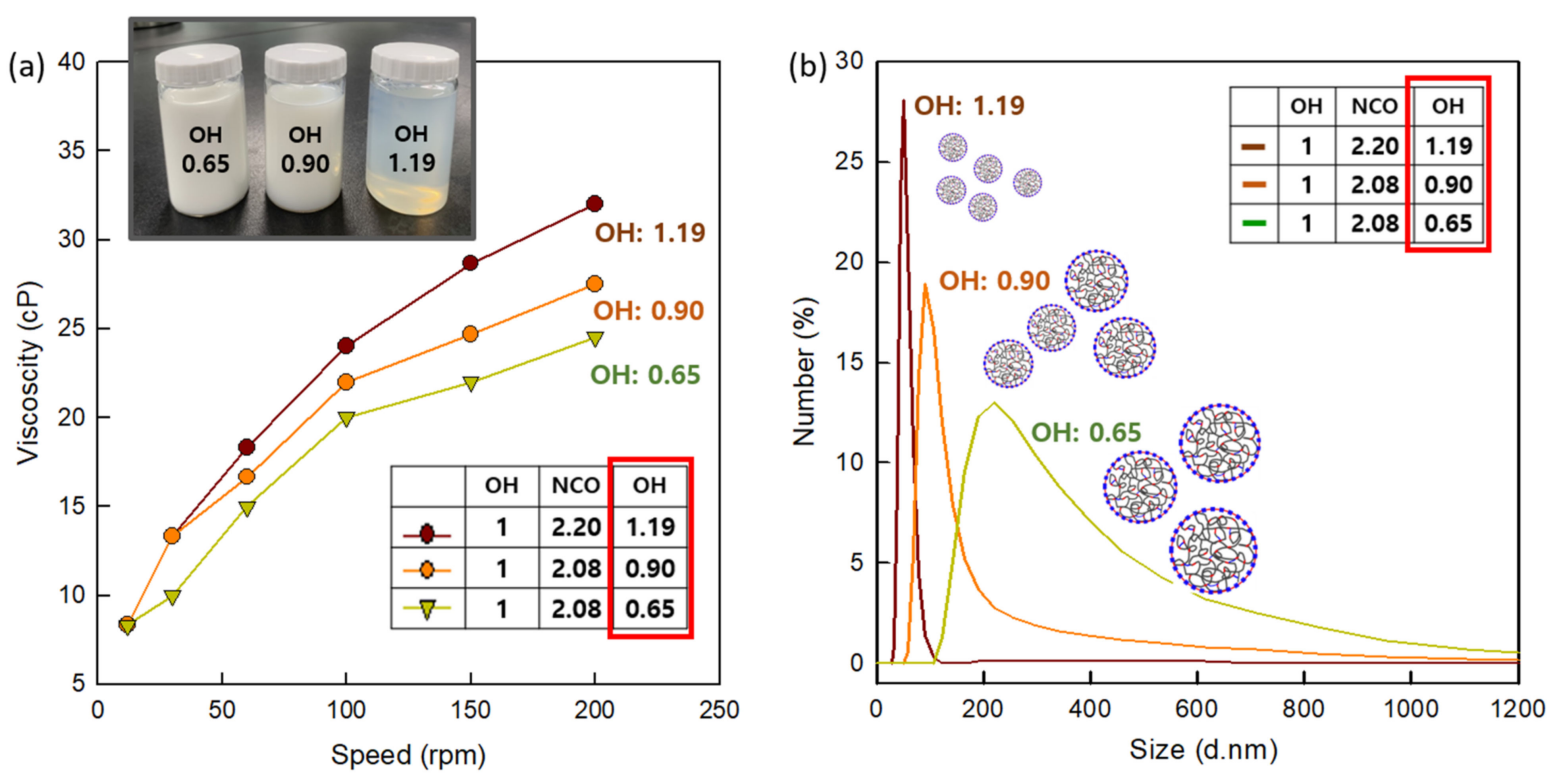
(**a**) Viscosity and (**b**) size of the synthesized CWPU dispersions with different ratio of OH/NCO/OH.

In order to analyze the effect of the emulsifier, castor oil-based anionic waterborne polyurethane (CWPU) dispersions were prepared with three kinds according to the ratio of the emulsifier, 1/2.08/0.65, 1/2.08/0.9 and 1/2.2/1.19. The ionic groups of the emulsifier form hydrophilic groups in the polyurethane (PU) backbone chain, which also provides surface negative charges for hydrophobic PU segments to disperse in water [22]. Considering the overall chemical structure of polyurethane, since the emulsifier constitutes the hard segment, the amount of the hard segment increases according to the amount of the emulsifier, resulting in an increase in the properties of the formed film. Therefore, it could be expected that the emulsifiers play an important role in influencing the properties of polyurethane dispersions and the resulting films. The effect of the amount of emulsifier on particle size and viscosity was analyzed (Figure 1), the contents of hard segment (HS) in CWPU with 1/2.08/0.65, 1/2.08/0.9 and 1/2.2/1.19 are 49.4%, 51.39%, and 52.49%, respectively. It indicates that the viscosity of the fabricated CWPU according to the various ratio of the emulsifier increases with rate of speed due to triglyceride structure of castor oil (Figure 1a). It is considered that the viscosity of CWPU dispersions made of soft segments with triols increases, since the chains are not released and become more agglomerated by rotation. In general, their viscosity raises with amount of emulsifier attributed to the effect of CWPU particle size. As the average particle size of CWPU decreases, the viscosity of the dispersion increases because of the number of particles and effective surface interaction [27]. It is confirmed that the effect of the amount of emulsifier on particle size which is associated with that the OH molar ratio of DMBA decreases from 1.19 to 0.65, resulting in an increase of the average particle size from 70 nm to 280 nm (Figure 1b). The smaller in size and more in the number of particles, the more the dispersion becomes transparent. That seems to be due to the increase in the ionic center content of the ionomer dispersion. In the case of water-based polyurethane dispersion, generally, it is known that as the hydrophilicity increases, the particle size decreases [28]. That is, the increase in the carboxylic acid hydrophilic group of the anionic WPU means that the increased added amount of DMBA augments the hydrophilic groups in the HS of the entire CWPU. As a result, when it is dispersed in water, the particle size of CWPU is reduced, leading to a transparent and viscosity-increased dispersion. Here, we decide to use CWPU with 1/2.08/0.9 of OH/NCO/OH for a further step. This is because 1/2.2/1.19 is the smallest size of the average particle size (70 nm), however it has high viscosity due to poor dispersibility. In addition, although 1/2.08/0.65 has a low viscosity, it is well-known that the particle size has an important bearing on stability of the dispersion and small particle size (<200 nm) shows storage stable and a high surface energy [29].

### 3.2. Characteristics of the CWPU/RCNs Nanocomposites Dispersions and Films According to the Step of Adding RCNs

**Figure 2:**
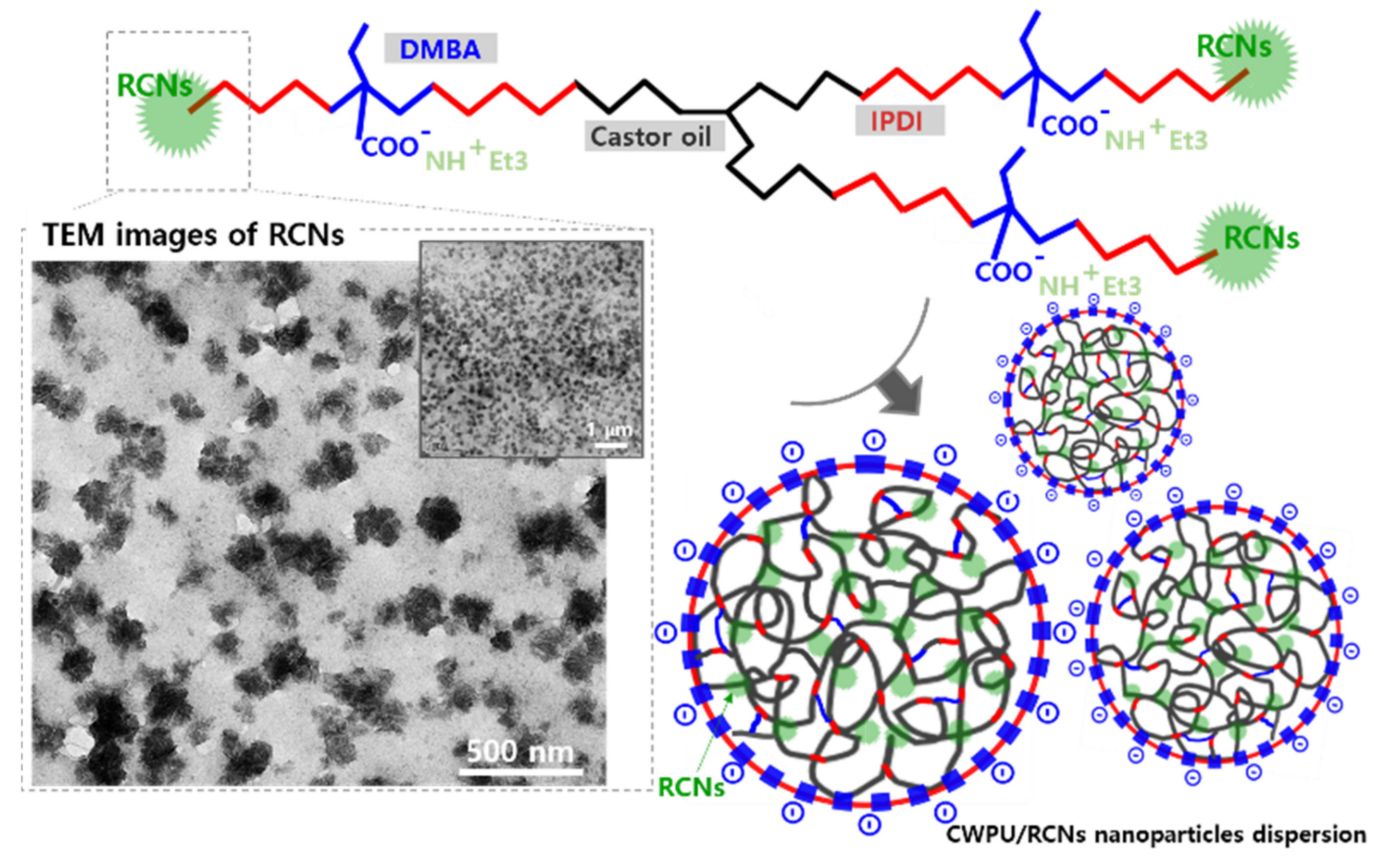
The structure of the fabricated CWPU/RCNs nanocomposites and TEM (inset figures) images.

CWPU/RCNs nanocomposites were prepared using the emulsifier ratio mentioned above, 1/2.08/0.9 of OH/NCO/OH (Figure 2). RCNs are complexed with CWPU dispersion, accordingly forming interpenetrating networks which are attributed to urethane groups (NHCOO) formed between NCO of IPDI and OH of cellulose in situ. Furthermore, in addition to urethane bonds, secondary physical bonds including hydrogen bonds also have a critical effect on the mechanical performance of the fabricated nanocomposite films. 

In this experiment, we studied how the properties of the resulting nanocomposite films were affected depending on when RCNs are added. As mentioned in the paragraph above, CWPU with 1/2.08/0.9 of OH/NCO/OH was used as a sample to prepare a nanocomposite dispersions and films by adding RCNs particles. From here on, it was referred to as CWPU.

**Figure 3:**
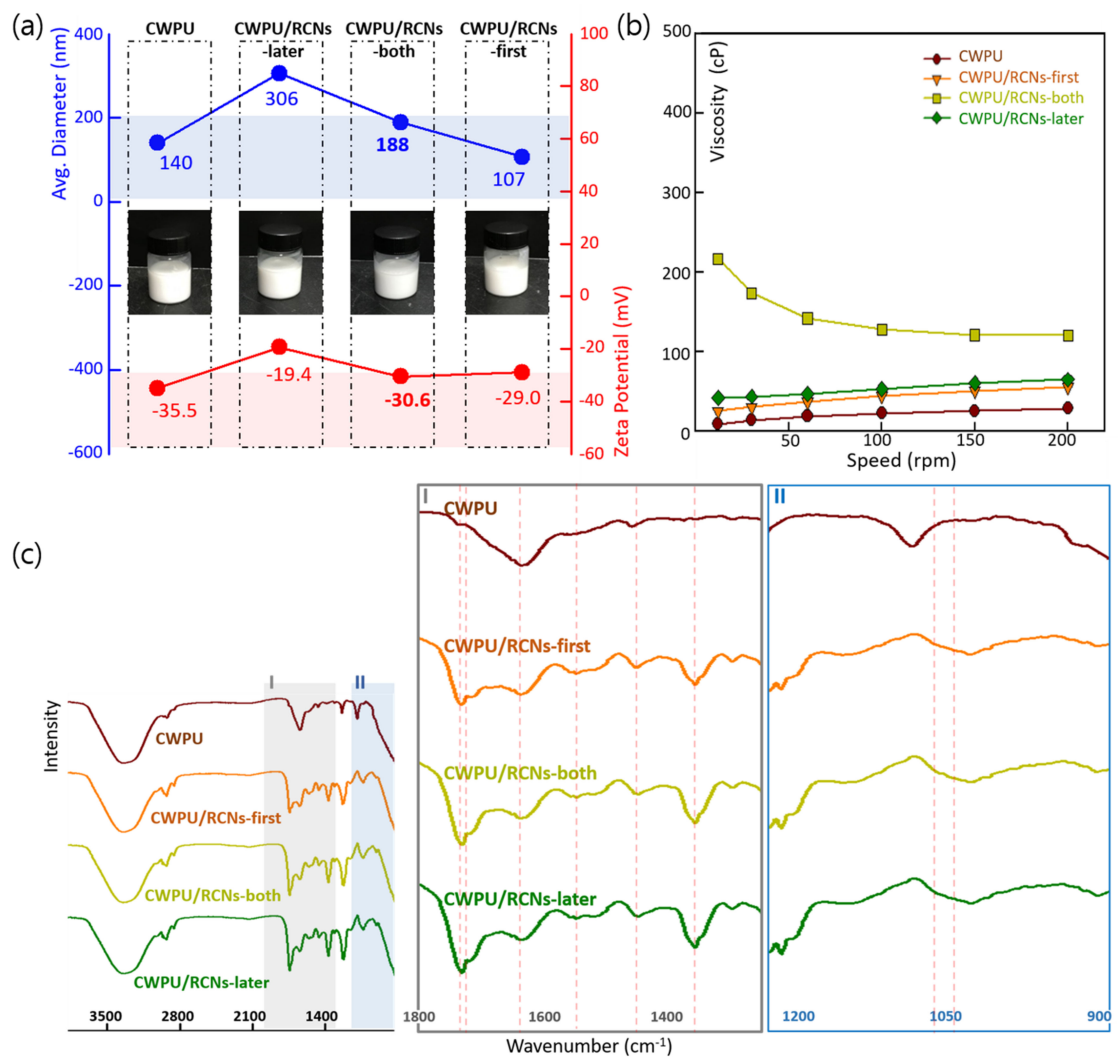
(**a**) Particle size and zeta potential, (**b**) viscosity, and (**c**) FT-IR spectra of CWPU and CWPU/RCNs dispersions according to the step of adding RCNs.

The particle size and zeta potential with their appearances, viscosity versus speed, and chemical distribution of CWPU and CWPU/RCNs nanocomposites dispersions according to the step of adding RCNs are provided (Figure 3), it is observed that the particle size and zeta potential are altered to the RCNs adding step where the absolute value of particle size has the similar trend as the zeta potential (Figure 3a). H. Liang et al. [30] explained that when it comes to the correlation between particle size and zeta potential, a thinner electric double layer reduces the electrostatic repulsion between the WPU dispersed particles, making the particle size larger. It is observed that CWPU exhibits negative zeta potential of −35.5 mV indicating the stability of the emulsion. Actually, no precipitation occurred even after centrifugation at 3000 rpm for 30 min. In case of CWPU/RCNs-both and CWPU/RCNs-later, the particle size is increased and the absolute value of zeta potential is decreased, which is thought to be due to a correlation between urethane (NHCOO) linkage or hydrogen bonds between CWPU and RCNs, accordingly to the length of chains. However, the absolute value of zeta potential still remains above 30 in case of CWPU/RCNs-both, which means that as the thickness of the hydrated double electric layer in the CWPU/RCNs nanocomposite dispersions increases, their stability also increases. This leads to excellent mechanical and thermal stability. Whereas, it was analyzed that the particle size of CWPU/RCNs-first was smaller than that of CWPU, it seems that CWPU/RCNs nanocomposite particle could not be formed. This is also consistent with the FTIR analysis (this phenomenon also appears in viscosity analysis or IR analysis.) In results of viscosity, the viscosity of CWPU, CWPU/RCNs-first, and CWPU/RCNs-later are increased due to aggregation with rotation, whereas that of CWPU/RCNs-both showed the opposite trend (Figure 3b). As RCNs are added, the chain lengthens and the size tends to increase, and as the rotation speed increases, the electrical double layer was loosened and particle orientation in the rotation direction becomes easier and the viscosity decreases. Compared to CWPU without RCNs, the characteristic absorption peaks at around 1731, 1365, 1317, 1196, and 1055 cm^−1^ appear stronger, which are associated with the urethane hydrogen-bonded carbonyl groups (C=O), cellulose CH stretching band, cellulose CH_2_ wagging band, cellulose C-C ring vibration, and hydrogen bond [26], respectively (Figure 3c and Table 2). It is the basis that CWPU/RCNs nanocomposites have good dispersibility, which was demonstrated by Pei et al. [31]. It is considered that the absorption of the carbonyl group (C=O) is due to the reaction between hydroxyl group and isocyanate group of cellulose. The stretching vibration of C=O group derived from free carbonyl groups (urethane) at 1731 cm^−1^, hydrogen bonded carbonyl groups (urethane) at 1705 cm^−1^, and hydrogen boned carbonyl groups (urea) at 1630 cm^−1^, respectively. Additionally, the broad peak of 3070–3750, centered at 3325 cm^−1^ for CWPU corresponds to the stretching vibration of NH bond. The band at 1549 cm^−1^ is attributable to the bending vibration of NH bond which is characteristic of the urethane group [32,33].

The peak intensity and area of the hydrogen bond of CWPU was in the order of both = later > first. The both and later groups were effective methods for in situ polymerization of CWPU/RCNs. The first seems not effective to synthesize the nanocomposite with RCNs, which estimated the intensity of related RCNs bands at 1365, 1196 and 1055 cm^−1^. From the CWPU/RCNs manufacturing process point of view, the ’both’ method is simpler than the ’later’ method. This is because the RCNs addition process can be contained with water adding step concurrently. 

**Figure 4:**
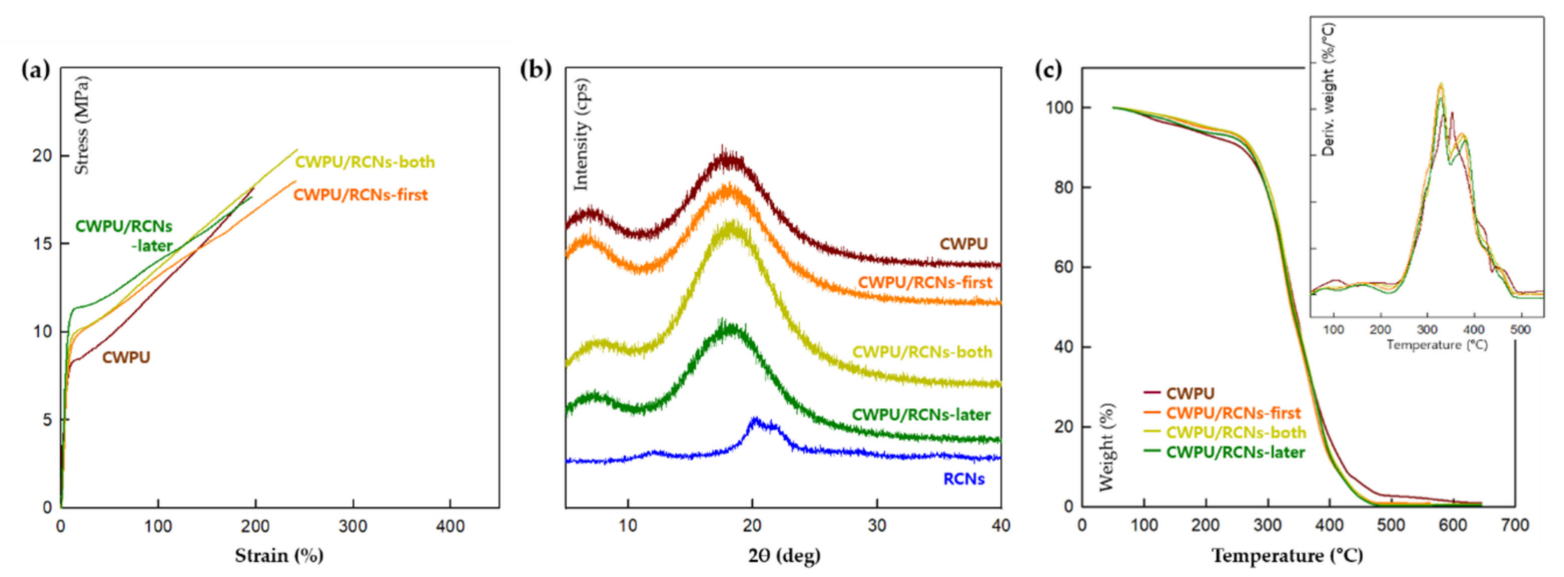
(**a**) Stress-strain curves, (**b**) XRD pattern, (**c**) TGA dynamic thermograms of CWPU and CWPU/RCNs nanocomposite films according to the step of adding RCNs.

Tensile, X-ray and thermal properties were analyzed to study the characteristics of the prepared CWPU/RCNs nanocomposite films according to the step of adding RCNs (Figure 4). The stress and Young’s modulus of CWPU/RCNs nanocomposites are higher than that of neat CWPU due to increasing contents of hard segments, which induced enhancing mechanical properties of the CWPU/RCNs nanocomposite films (Figure 4a and Table 3). Not only stress, but also strain is increased, which is because the RCNs are contained in hard segments. Since the crystallinity of RCNs is only 28.2% as shown in the X-ray result, it seems to allow the CWPU hard segments to soften, resulting in the increase in strain with stress. However, in spite of using the same hard segments, the results may depend on the step of adding the RCNs. From their stress and toughness standpoint, CWPU/RCNs both samples are superior compared to other steps, which is advantageous for their use as elastomers. It is well-known that cellulose crystals exist in crystalline I, II, III, and IV form [34] as shown in the X-ray curves of CWPU and CWPU/RCNs nanocomposite films (Figure 4b). The prepared RCNs show slight crystallinity observed at 2θ = 20° and 21.5°, corresponding to the (101) and (021) crystal planes of cellulose II [3], and a broad diffraction peak at 2θ = 19.8° of CWPU is responsible for the amorphous nature of WPU [35]. However, from the introduction of RCNs into the CWPU matrix, the diffraction peaks of RCNs overlapped and strengthened around 21.5°, which appears prominently at CWPU/RCNs both samples. The initial degradation temperature of CWPU/RCNs nanocomposite films is increased by the addition of RCNs, and the main degradation temperature (350~400 °C) is higher compared to that of neat CWPU films as shown in derivative thermogravimetric graph, followed by later > both > first (Figure 4c). It is determined that the thermal resistance by the addition of RCNs is improved due to the hard segment formation of a structure in the CWPU/RCNs nanocomposite films. 

### 3.3. Characteristics of the CWPURCNs Nanocomposites Dispersions and Films According to RCNs Contents

**Figure 5:**
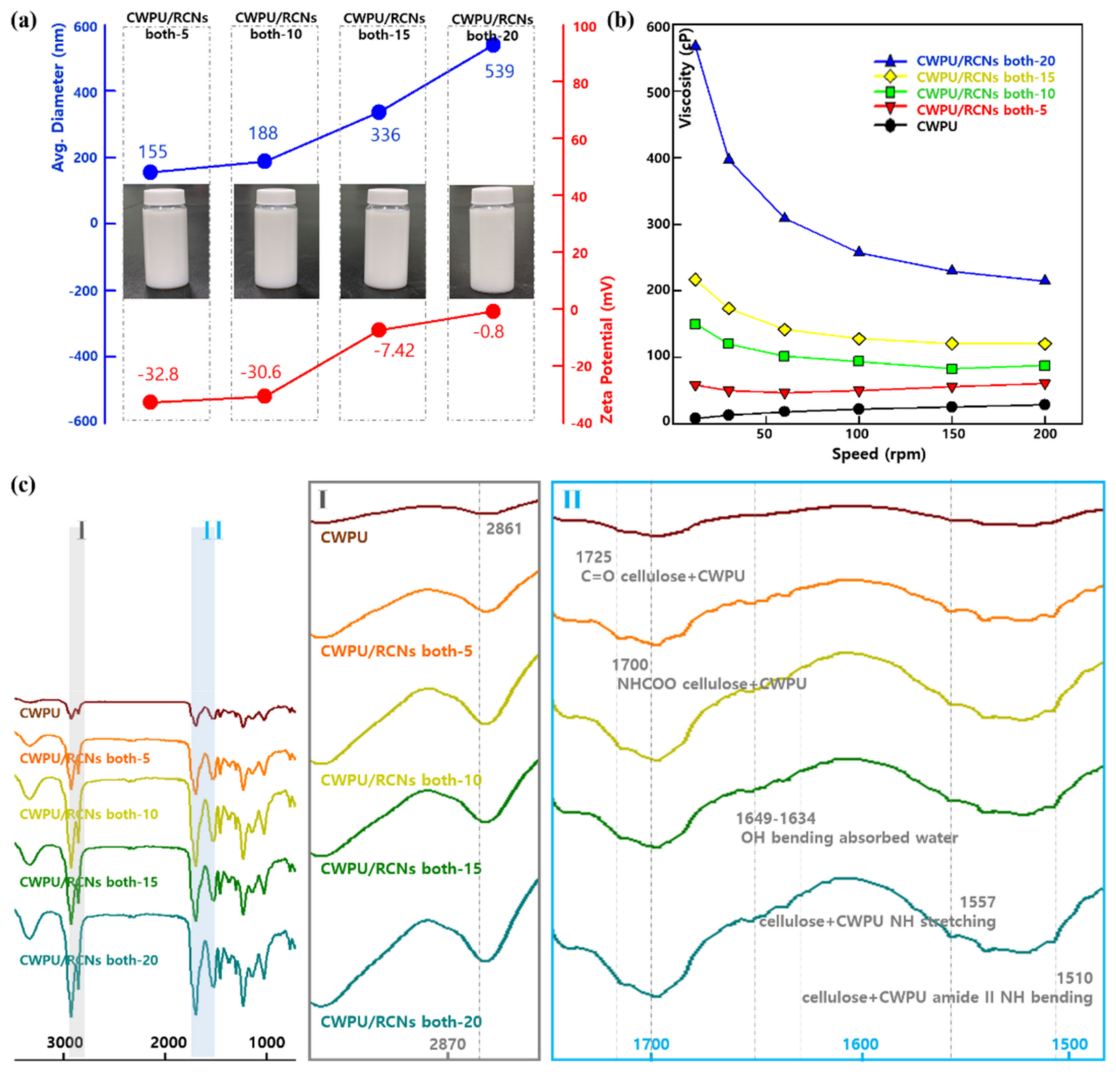
(**a**) Particle size and zeta potential measurement, (**b**) viscosity properties, and (**c**) FTIR spectra of the prepared CWPU and CWPU/RCNs nanocomposite dispersions according to RCNs contents.

The thinner electrical double layer reduces the electrostatic repulsive forces between the CWPU particles due to the addition of RCNs into the hard segment, which in turn creates larger and larger particle sizes. The value of zeta potential has a similar trend to the particle size of the CWPU/RCNs. The zeta potential varied from −32.8 to −0.8 mV and the mean particle size increased from 155 to 539 nm (Figure 5a). As the RCNs contents were further increased, the absolute value of zeta potential was found to decline gradually. Agglomeration of the CWPU/RCNs nanocomposites occurs when the individual particles unite by weak inter-particle interaction, electrostatic attraction and Van der Walls forces [36,37]. Additionally, the value of viscosity increases according to RCNs contents due to an interaction between CWPU and RCNs particles (Figure 5b). While the CWPU dispersion shows as agglomerated by rotation due to the triol-structure, the CWPU/RCNs nanocomposite dispersions exhibit pseudo plastic behavior due to the addition of cellulose segments in the hard segment of CWPU, which is prominent as the RCNs contents increase. The FTIR curve shows the characteristic peak of the prepared CWPU/RCNs nanocomposite dispersions according to RCNs contents (Figure 5c and Table 4). The broad peak at 3100–3550 cm^−1^ corresponds to a stretching vibration of –NH and –OH group, whereas the stretching vibration peak of CH_2_ is located at 2800–3000 cm^−1^. The CH_2_ stretching vibration peak of cellulose is located at 2861 cm^−1^ and represents its strengthening with the RCNs contents. The peaks referring to the CWPU/RCNs nanocomposite are 1725, 1700, 1557, and 1510 cm^−1^, and these peaks represent C=O, NHCOO, NH, and amide II NH, respectively, which appear by forming a nanocomposite with RCNs. The intensity of these peaks increased as the RCNs content increased, the peaks in 1649–1634 cm^−1^ corresponding to OH bending of absorbed water due to cellulose also increase with the RCNs content. 

**Figure 6:**
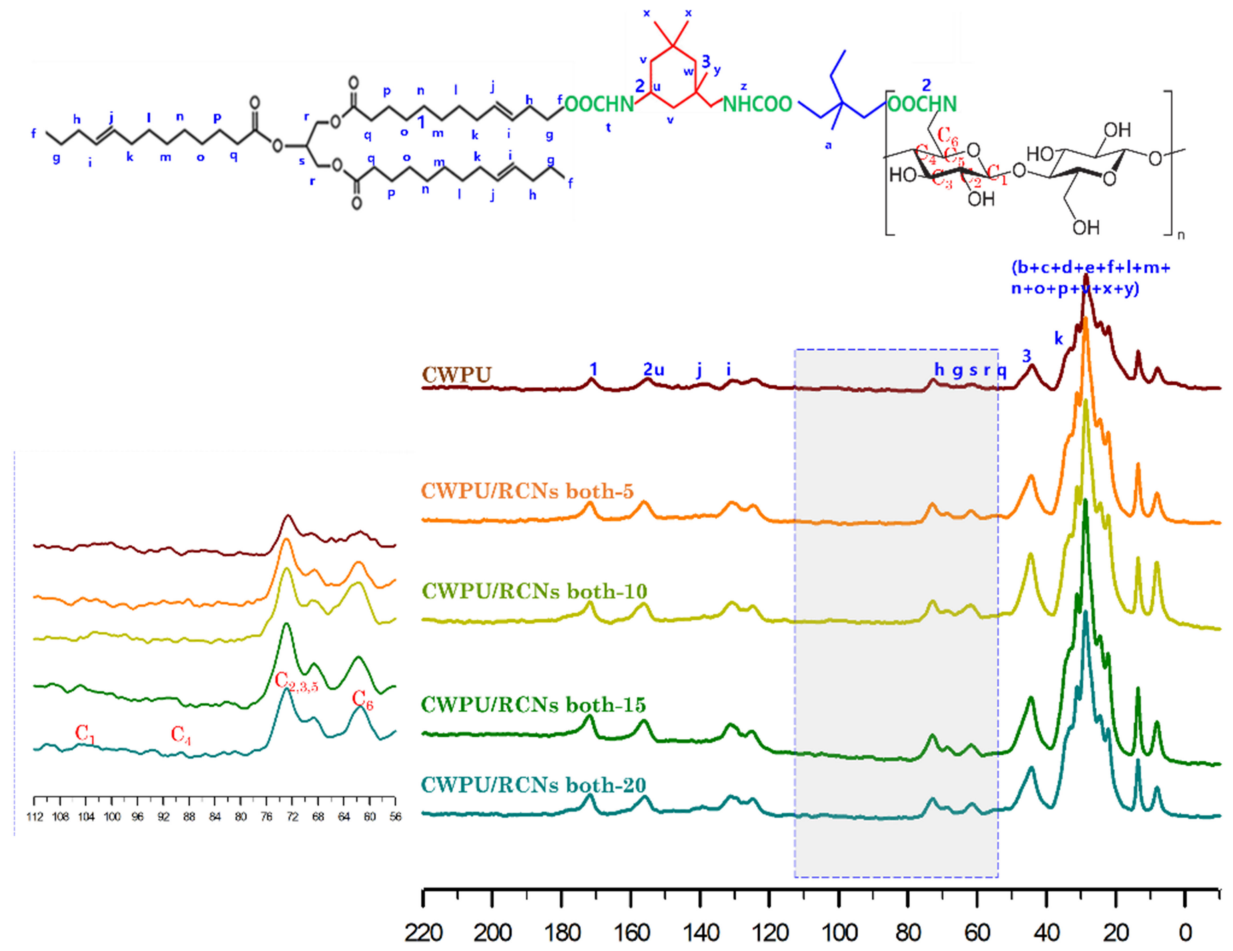
Solid state ^13^C NMR spectra of the prepared CWPU and CWPU/RCNs nanocomposite films according to RCNs contents.

It can be determined the presence of carbon in cellulose in the CWPU/RCNs nanocomposite films as well as the structure of the neat CWPU from solid state ^13^C NMR spectroscopy (Figure 6). The signals at 62.5 denote the C6 atom, while the peaks at 72.9–75 ppm describe the C2, C3, and C5 carbons of cellulose. In addition, C4 and C1 display 88.9 and 104–106 ppm, respectively. Depending on the overall crystallinity of cellulose, these peak shapes can vary significantly; RCNs with low crystallinity were used for preparing CWPU/RCNs nanocomposite films. A higher resolution and sharper peaks usually implies a higher crystallinity, which however can be influenced by chemical treatments such as synthesizing with bio-based derivates [38,39,40]. The peak was not clear nor appeared in pure CWPU, whereas the typical peaks assigned to cellulose appeared clearly, confirming the existence of RCNs in the CWPU/RCNs nanocomposite films. 

**Figure 7:**
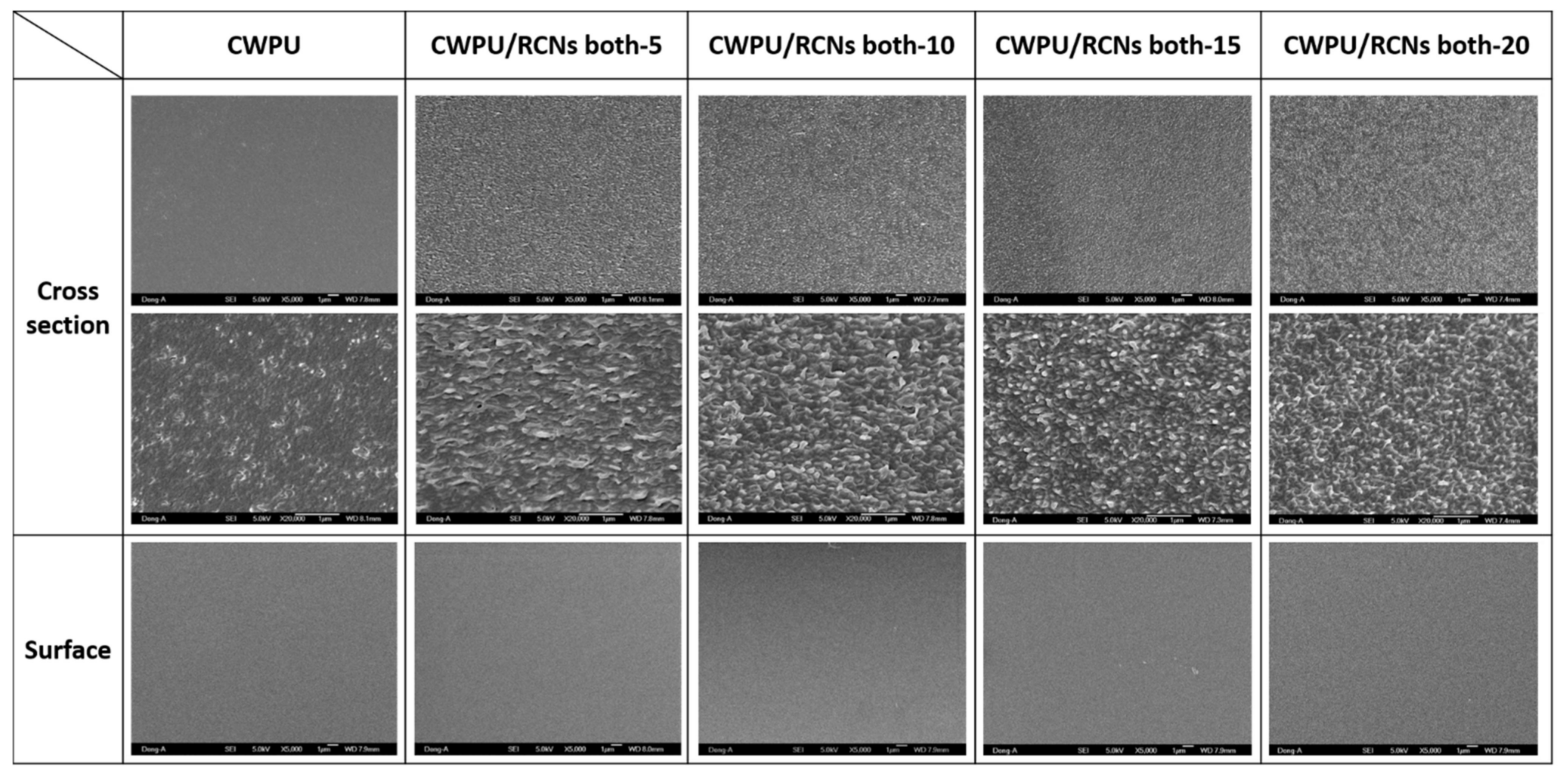
Surface and cross-section scanning electron microscopy (SEM) images of the CWPU and CWPU/RCNs nanocomposite films according to RCNs contents.

The SEM images of the frozen-fractured cross-sections and the surfaces of the prepared CWPU/RCNs nanocomposite films were examined to observe the effect of various RCNs contents in the CWPU/RCNs nanocomposite films (Figure 7). The RCNs were identified as white dots in the fractured surfaces of the nanocomposite films. Roughening showed homogeneous distribution all over the fractured surface, indicating good distribution of RCNs in the nanocomposite films. Their frozen-fractured surface appears to be rugged, but shows an even texture. It is observed that the cross section showed a rougher surface with an increase in the content of RCNs and is evenly distributed throughout. As is well known, homogeneous dispersion of RCNs integrated with strong interactions are indispensable for improving the mechanical stability of the CWPU/RCNs nanocomposite films [41].

**Figure 8:**
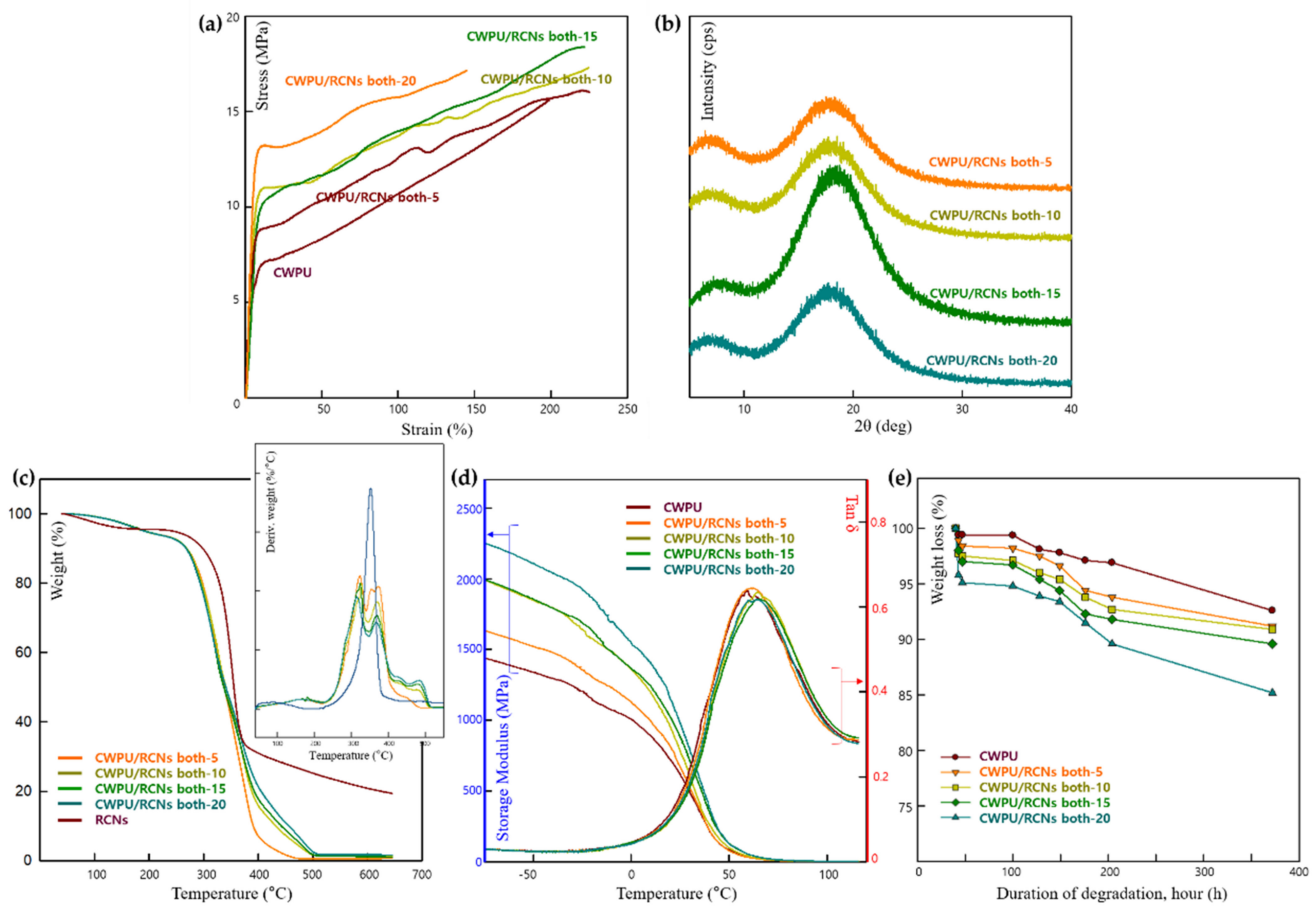
(**a**) Stress-strain curves, (**b**) XRD patterns, (**c**) TGA dynamic thermograms, (**d**) storage moduli and tan as a function of temperature, and (**e**) dependence of the weight loss as a function of time during the cellulase degradation of the CWPU and CWPU/RCNs nanocomposite films according to RCNs contents.

The addition of RCNs to the CWPU matrix has a strong effect on various characteristics of the nanocomposite films, such as mechanical, thermal, and degradation properties (Figure 8 and Table 5). In general, with increasing filler contents, Young’s modulus of the prepared nanocomposite films increases due to relatively high chemical crosslinking that can restrict the chain mobility and increase the intermolecular attraction between hard segments, whereas elongation at break tends to decrease. In this study, the tensile strength and Young’s modulus were increased for CWPU/RCNs both 15–20, however the elongation at break was increased for CWPU/RCNs both 5–10 contrary with the maximum value of 58.8 ± 3 kgf∙mm to the above (Figure 8a). The enhancement of toughness in the CWPU/RCNs nanocomposite films benefits from the good interfacial interaction between RCNs and CWPU and the net structure with reinforced connection, which can transfer stress under external stress effectively. On the other hand, in the CWPU/RCNs both 15 and 20, the toughness and even tensile strength were reduced by incorporating RCNs, which is similar to the research of Zhang et al. This decrease can be mainly attributed to the interruption in the original continuous structure of CWPU chains with the addition of excessive RCNs. In addition, due to the agglomeration of RCNs, the increase in the stress concentration point causes defects in the prepared films, which in turn eventually leads to a decrease in maximum tensile strength, elongation at break, and toughness. As the content of RCNs increased, the intermolecular attraction between hard segments increased, resulting in an increase in Young’s modulus and elongation at break due to the structure of RCNs with low crystallinity. That is, it indicates that the prepared CWPU/RCNs nanocomposite films have become harder and tougher. Wide angle XRD was employed to determine the effect of RCNs on the mechanical properties of CWPU/RCNs nanocomposite films. All CWPU/RCNs nanocomposite films show similar XRD patterns, introducing RCNs into the CWPU matrix resulted in the two characteristic diffraction peaks of 2θ = 20° and 21.5° peaks, corresponding to the (101) and (021) crystal planes of cellulose II, are pronounced as increasing RCNs contents (Figure 8b). It could be a factor in the higher mechanical and thermal properties of CWPU/RCNs nanocomposites films with increasing RCNs contents. RCNs plays an important role in determining thermal stability of the critical characteristic for the nanocomposite films. According to the thermogravimetry (TG) and derivative weight change, there are not differences between each CWPU/RCNs sample in initial region, whereas they show a distinct difference above 340 °C (Figure 8c). Before 340 °C, the rate of weight decrease is rapid, but after that, the decrease seems to be sluggish as the RCNs contents increase. This is because RCNs has greater thermostability than CWPU. The residue ash content of the CWPU/RCNs samples increases in proportion to RCNs contents.

From the information related to the viscoelastic properties such as stiffness and energy dissipation of the CWPU obtained different amounts of RCNs (Figure 8d), there is a prominent increase in the modulus of CWPU matrix with the incorporation of RCNs over the entire region compared to a neat CWPU matrix. The value of storage modulus is increased by the addition of RCNs in CWPU, this is due to interactions between CWPU and RCNs via intercalating in WPU hard segments that allowed a greater degree of stress transfer at the interface. The ratio of the loss modulus to the storage modulus is measured as the mechanical loss factor, tan δ. The damping properties give the balance between the elastic phase and viscous phase in the CWPU/RCNs nanocomposite structure. In the prepared nanocomposites, damping is influenced by the incorporation of RCNs, the tan peak is indicative of the structure and properties of nanocomposites. It is observed that the tan δ peak height decreases with the increase in RCNs contents, this seems due to the restriction of movement of polymer molecules by the incorporation of RCNs [42]. It also indicates outstanding interfacial adhesion. The addition of RCNs makes the tan δ peak shift to the right, from 59.5 to 65.4 °C, which can be attributed to the declined mobility of the chain with increasing RCNs. Cellulases cleave glucosidic bonds by using acid-based catalysis. It is confirmed that the mass loss of all samples increases with time due to degradation of RCNs by celluase enzyme, from the analysis of the mass loss of CWPU/RCNs after degradation in 2 wt% cellulase (Figure 8e). During 470 h of degradation, the CWPU/RCNs both-20 had the highest degradation rate, which seems to be also attributed from the CWPU made from castor oil, which in turn can be affected by the enzymes.

## 4. Conclusions

In summary, we have presented here a new idea for in situ synthesizing of environmentally friendly bio-polyurethane nanocomposite dispersions and films with high toughness and biodegradability. In our approach, polymerization conditions for waterborne polyurethane are established without using catalysts which are harmful to the human body or ethyl methyl ketone (MEK) to reduce the viscosity of the prepolymer during the reaction for an eco-friendlier process. Moreover, regenerated cellulose nanoparticles (RCNs) are used as natural chain-extenders in order to enhance the biodegradability and mechanical properties of the prepared nanocomposite films. The RCNs used in this study have a lower crystallinity compared to cellulose nanocrystals (CNCs), enabling the development of nanocomposite films with high toughness without interfering with the elastomeric properties of polyurethane. In this study, we drew the most suitable and appropriate polymerization conditions through sophisticated experiment conditions such as an emulsifier ratio, the adding sequence of RCNs into prepolymer, and RCNs contents for the development of environmentally friendly bio-polyurethane nanocomposite dispersions and films with high toughness and biodegradability; it was indicated that the elasticity and mechanical properties increased using RCNs with low crystallinity. The fabricated CWPU/RCNs nanocomposite films showed high toughness of 58.8 ± 3 kgf∙mm and elongation at break of 240 ± 20%. Moreover, depending on the molar ratio of NCO/OH, the size of the polyurethane particles is variously controlled, allowing to prepare dispersions with various transmittances. This has a lot of potential for use in various applications, such as coating agents, biosensor substrate, general purpose tubing, furniture, bedding, thermal insulation, apparel, flooring and so forth, by offering both elasticity and high toughness to environmentally-friendly CWPU dispersions. We believe that our findings not only open a significant path toward more environmentally friendly elastomers with biodegradability, but also provide a bio-elastomer design concept for fabricating industrial elastomers with mechanical and thermal properties. 

## Figures and Tables

**Table 1:** Compositions of CWPU and CWPU/RCNs nanocomposites.

Sample	Molar Ratio			
	OH (Castor Oil)	NCO (IPDI)	OH (DMBA)	RCNs Content (wt% of CWPU)
CWPU 0.65	1	2.08	0.65	-
CWPU 0.90	1	2.08	0.90	-
CWPU 1.19	1	2.20	1.19	-
CWPU/RCNs-5	1	2.08	0.90	0.18
CWPU/RCNs-10	1	2.08	0.90	0.37
CWPU/RCNs-15	1	2.08	0.90	0.58
CWPU/RCNs-20	1	2.08	0.90	0.76

**Table 2:** Curve-fitting results **of** FTIR peak assignments for CWPU and CWPU/RCNs dispersions according to the step of adding RCNs in Figure 3c.

Related Group	C=O Stretching of Urethane Group		Cellulose CH Stretching Band		Cellulose CH_2_ Wagging Vibration		Cellulose C-C Ring Vibration		Cellulose C-O-C Ring Vibration		Hydrogen Bond	
Location (cm^−1^)	1731		1365		1317		1196		1062		1055	
Items	Intensity	Area	Intensity	Area	Intensity	Area	Intensity	Area	Intensity	Area	Intensity	Area
Samples												
CWPU	3	125	0.9	10	1.08	19	0.45	3	0.05	0.04	1.6	6
CWPU/RCNs-first	23	781	11	189	2.56	39	6	234	4	164	8	220
CWPU/RCNs-both	22	714	15	262	2.27	35	13	517	6	91	9	207
CWPU/RCNs-later	25	762	17	301	2.39	38	15	517	7	131	9	283

**Table 3:** Young’s modulus, tensile strength, toughness, and percentage elongation of CWPU and CWPU/RCNs nanocomposite films according to the step of adding RCNs in Figure 4a.

Sample	Hard Segment Content (wt%) ^1^	Young’s Modulus (MPa)	Tensile Strength (MPa)	Elongation at Break (%)	Toughness (kgf∙mm)
CWPU	51.35	124.5 ± 4.7	15.5 ± 3.2	190 ± 15	40.5 ± 2.2
CWPU/RCNs-first	52.23	134.5 ± 5.3	22.5 ± 2.4	230 ± 20	48.4 ± 2.7
CWPU/RCNs-both	52.23	139.6 ± 6.5	17.2 ± 3.2	240 ± 20	58.8 ± 3.0
CWPU/RCNs-later	52.23	144.3 ± 7.6	17.6 ± 2.7	196 ± 25	43.5 ± 3.4

^1^ Hard segment content = [mass of DMBA, IPDI, RCNs and TEA/mass of CO, DMBA, IPDI, RCNs and TEA] × 100.

**Table 4:** Curve-fitting results **of** FTIR peak assignments for CWPU and CWPU/RCNs nanocomposite dispersions according to RCNs contents in Figure 5c.

Related Group	CH_2_ Cellulose		C=O Cellulose + CWPU		NHCOO Cellulose + CWPU		OH Bending Absorbed Water		Cellulose + CWPU NH Stretching		Cellulose + CWPU Amide II Bending	
Location (cm^-1^)	2861		1732		1700		1649		1557		1510	
Items	Intensity	Area	Intensity	Area	Intensity	Area	Intensity	Area	Intensity	Area	Intensity	Area
Samples												
CWPU	3.8	76	7.08	273	11.9	3520	4.07	102	4047	70	5.92	268
CWPU/RCNs both-5	11.75	239	18.72	403	25.78	743	9.73	123	10.08	117	11.14	130
CWPU/RCNs both-10	17.46	361	25.25	957	35.42	1289	13.35	216	13.74	237	14.75	354
CWPU/RCNs both-15	19.44	396	29.80	991	42.85	1426	16.12	300	16.86	314	17.28	308
CWPU/RCNs both-20	22.87	481	33.18	1259	47.68	1678	18.13	311	18.89	373	19.41	349

**Table 5:** Young’s modulus, tensile strength, toughness, T_g_, and percentage elongation of CWPU and CWPU/RCNs nanocomposite films according to RCNs contents in Figure 8.

Sample	Hard Segment Content (wt%)^1^	Young’s Modulus (MPa)	Tensile Strength (MPa)	Elongation at Break (%)	Toughness (kgf∙mm)	T_g_ from tan δ (°C)
CWPU	51.35	124.5 ± 4.7	15.5 ± 3.2	190 ± 15	40.5 ± 2.2	59.5
CWPU/RCNs both-5	51.81	133.6 ± 5.3	16.0 ± 2.5	224 ± 20	47.4 ± 3.3	62.5
CWPU/RCNs both-10	52.23	139.6 ± 6.5	17.2 ± 3.2	240 ± 20	58.8 ± 3.0	61.4
CWPU/RCNs both-15	53.04	150.3 ± 5.1	18.5 ± 2.3	222 ± 15	49.4 ± 3.1	64.8
CWPU/RCNs both-20	53.82	155.6 ± 3.6	17.1 ± 2.2	144 ± 13	35.8 ± 3.5	65.4

^1^ Hard segment content = [mass of DMBA, IPDI, RCNs and TEA/mass of CO, DMBA, IPDI, RCNs and TEA] × 100.

## Data Availability

Data is available from the corresponding author on reasonable request.
